# Psychoactive substances and the political ecology of mental distress

**DOI:** 10.1186/1477-7517-9-4

**Published:** 2012-01-18

**Authors:** Sunil K Aggarwal, Gregory T Carter, Craig Zumbrunnen, Richard Morrill, Mark Sullivan, Jonathan D Mayer

**Affiliations:** 1 Department of Physical Medicine and Rehabilitation, New York University, Rusk Institute of Rehabilitation Medicine, 400 E 34th St, New York, NY 10016 USA; 2 Department of Rehabilitation Medicine, University of Washington, Seattle, Washington. 1959 NE Pacific Street, Box 356490, Seattle, WA 98195 USA; 3 Department of Geography, University of Washington, Seattle, Washington, Box 353550, Smith Hall 408, Seattle, WA 98195 USA; 4 Department of Psychiatry and Behavioral Sciences, University of Washington, Seattle, Washington. Box 356560, 1959 N.E. Pacific St, Seattle, WA 98195 USA; 5 Department of Bioethics and Humanities, University of Washington, Seattle, Washington. Box 357120, 1959 N.E. Pacific St, Seattle, WA 98195 USA; 6 Department of Epidemiology, University of Washington, Seattle, Washington. Box 357236, 1959 NE Pacific Street, Seattle, WA 98195 USA; 7 Department of Global Health, University of Washington, Seattle, Washington. Harborview Medical Center, Box 359931, 325 9th Avenue, Seattle WA 98104; 8 Division of Allergy and Infectious Diseases, Department of Medicine, University of Washington, Seattle, Washington. Box 356423, Seattle, WA 98195 USA; 9 Department of Family Medicine, University of Washington, Seattle, Washington. Box 356390, 1959 NE Pacific Street, Seattle, WA 98195 USA; 10 Department of Health Services, University of Washington, Seattle, Washington. Box 357660, 1959 NE Pacific Street, Seattle, WA 98195 USA

## Abstract

The goal of this paper is to both understand and depathologize clinically significant mental distress related to criminalized contact with psychoactive biotic substances by employing a framework known as critical political ecology of health and disease from the subdiscipline of medical geography. The political ecology of disease framework joins disease ecology with the power-calculus of political economy and calls for situating health-related phenomena in their broad social and economic context, demonstrating how large-scale global processes are at work at the local level, and giving due attention to historical analysis in understanding the relevant human-environment relations. Critical approaches to the political ecology of health and disease have the potential to incorporate ever-broadening social, political, economic, and cultural factors to challenge traditional causes, definitions, and sociomedical understandings of disease. Inspired by the patient-centered medical diagnosis critiques in medical geography, this paper will use a critical political ecology of disease approach to challenge certain prevailing sociomedical interpretations of disease, or more specifically, mental disorder, found in the field of substance abuse diagnostics and the related American punitive public policy regimes of substance abuse prevention and control, with regards to the use of biotic substances. It will do this by first critically interrogating the concept of "substances" and grounding them in an ecological context, reviewing the history of both the development of modern substance control laws and modern substance abuse diagnostics, and understanding the biogeographic dimensions of such approaches. It closes with proposing a non-criminalizing public health approach for regulating human close contact with psychoactive substances using the example of cannabis use.

## Giving 'Substances' substance and place

'Substance' is a shorthand term used in common parlance for '*psychoactive *substance', a pharmacologically active, consumable material, usually self-administered, that can reliably have, among other physiological effects, a discernible impact on one's mood, emotions, feelings, sensations, perceptions, and/or thinking. For the last century, consumption of a select group of psychoactive substances has been a matter of pressing political concern for modern State bureaucracies, and in that time all manner of popular conceptions concerning substance use, abuse, dependence, and addiction have had ample opportunity to be race-baited, red-baited, even gay-baited, chauvinistically slanted, politicized, inflated, and conflated due to a variety of cultural-historical reasons such as scapegoating, xenophobia, and 'culture wars' over the years, which an extensive literature has documented (see, for example:[1-3]). Nosology and diagnostics for substance-related mental disorders developed in health professional social circles and codified in standard psychiatry manuals have similarly shifted over time, with earnest attempts made in recent years at their summary de-politicization by mental health professionals and 'drugabuseologists'. But notwithstanding these efforts at putative 'scientific sanitization', this paper argues that long-hardened commitments to the normalized ideology of pharmacologicalism, eloquently described by DeGrandpre [4] as providing "a scientific foundation for the moral ordering of drugs" (p. 27), as in the good vs. bad/angel vs. demon/legal vs. illegal psychoactive substance dichotomies enshrined in high-level public policy, have uncritically been allowed to take root in medical diagnostic screening criteria for substance-related mental disorders. Under the current official diagnostic nosology, codified in the internationally used fourth edition of the American Psychiatric Association's Diagnostic and Statistical Manual for Mental Disorders (DSM-IV) [5], when a person engages in a pattern of substance use that leads to mental distress as manifested by their recurrent or year-long persisting substance possession-related legal problems, that person's substance use is seen as maladaptive, is summarily labeled pathologically self-abusive, and the individual is judged to be mentally disordered. While a fifth edition of DSM (DSM-V) is in the works which has proposed removal of diagnostic consideration of such legal problems, it is still undergoing revisions and not due for publication until May 2013 [6].

One may pause here and ask: what does any of this talk about the use of psychoactive substances have to do with a politics of the environment? Why address substance abuse diagnostic questions with a political ecology framework? Should this not be left to critical cultural studies of mental illness and psychiatry? That such questions even bubble to the surface is indicative of how successful the social mystifications that have arisen around psychoactive substance use have been in obscuring its basis in human relationships with the natural environment. Though often overlooked, many of the contested psychoactive substances in currency today (e.g., opium, coca, and cannabis) are botanicals found in the natural environment that evolved tens of millions of years ago. Very basic and well-defined human-environment relationships underpin the discovery, production, and consumption of all biotic psychoactive substances. Ultimately, it is argued here, addressing questions about human adaptation (or maladaptation) to psychoactive substance-replete natural environments, both at the societal and individual levels, is central for any clearheaded, scientific understanding of a given individual's substance use patterns and attendant mental distress that may be manifested, in order to judge whether that distress has a firm basis in psychopathology or not. The critical political ecology of disease approach is a suitable lens to use to address this question. Applying the rubric to such issues is not without precedent, as one veteran political ecologist, Paul Robbins, has called for a "political ecology of the drug trade" [7](p. 215), the beginnings of which have been sketched by Steinberg et al. [8,9].

Biotic substances usage can quite literally be grounded in precise and particular locales, yet the prevailing conceptions of substance use are anything-but grounded. It is a marvel that practical scholarship, to say nothing of policy, regarding a whole class of human-biota consumptive relations remains to this day to be wholly divorced from considerations of environmental ethics, co-evolution, and ecology. Take for example academic studies on problematic crack-cocaine consumption in American urban inner cities. While most studies of morbidity, morality, and social cost will examine social factors such as poverty, deprivation, and glamour surrounding problematic use and local distribution of the substance, rarely, if ever, will a study trace the crack-cocaine used by subjects to the thousands of pounds of coca leaves which were planted, grown, and harvested from which the cocaine alkaloid was extracted and later reacted with baking soda (sodium bicarbonate) and heat to produce crack 'rocks' that 'appear' in glass vials in the inner city for consumption. Nor will such studies earnestly question the normalized contraband status of the coca leaf botanical and the chemicals extracted from it, and what impacts that contraband status has on the chain of events linking problematic consumption of the substance in an urban inner city in the United States to, for example, cultivation practices in a Northern Peruvian rural village.

Understandably, the contraband status of such botanicals limits depth of inquiry. However, the problem could also be one of obfuscating terminology. While humans have lived and evolved within a world composed of material substance (and energy), psychoactive portions of this substance have come to be known, with no effort at semiotic clarity, simply as 'substances'. For the sake of rational grounding, let us divide these into biotic substances and abiotic substances. Biotic psychoactive substances are naturally occurring organisms that are an integral part of the biosphere and web of life in the same sense that any other terrestrially-evolved organisms are. They have unique secondary metabolite biochemical profiles that set them apart from other biota in that they contain chemicals that can robustly interact with endogenous systems of mood regulation, pleasure, muscle relaxation, and brain reward (among others) in humans and oftentimes other animals. They are the focus of this paper. As far as the abiotic substances are concerned, some, but not all of them, are unmodified or slightly modified concentrates of chemicals that were naturally biosynthesized in biotic substances. Others are novel products of the synthetic age.

## A political ecology of mental distress

Across cultures and throughout history and pre-history [10], human beings have known about biotic organisms living in their natural environments that, when intentionally ingested in whole or in part, could "stimulate, sedate, [palliate,] or elate" [11](p. 356). In the modern era, an arbitrary subgrouping of these living organisms, be they plants or fungi, along with the unique chemicals they produce and their related congeners, have become the locus of intense medical, public health, and international law enforcement focus. Today many who use biotic substances are vigorously pursued by law enforcement and punished by criminal justice systems using methods and tactics that increasingly undermine human dignity. For example, in the United States, the penalty for cannabis possession can be as severe as one year in federal prison for possession of any amount of marijuana, and up to five years in federal prison for growing one marijuana plant [12]. The death penalty is routinely used abroad and available to prosecutors in some cases in the US, multi-decadal mandatory prison sentences are routinely meted out to drug offenders, and other violations of drug offenders' privacy and family integrity are normalized. This, understandably, produces significant mental distress for those involved with these contraband biotic substances.

The ultimate stated purpose of the entire medico-legal apparatus positioned against these substances derives its final justification from a claim to act towards the 'prevention and control' of 'substance abuse' by individuals. A critical political ecology of disease perspective can shed light on the origins of substance use mental distress as manifested by biota possession legal problems and help address the central question: must this mental distress necessarily be viewed as a pathological sign of a maladaptive substance use pattern? After all, consumption, possession, or close proximity to biotic substances are all instantiations of particular human-environment relationships of close contact which, for now, are criminalized. When the latter fact is made manifest in one's life through encounters with some form of law enforcement, it is understandably mentally distressing considering the harsh punitive consequences that are allowed by law and routinely meted out.

On a personal note, the first author can attest to the reality of this mental distress, as he has personally experienced the mental distress of potential contraband biota possession-related legal problems and has been a target of a harassment episode where the threat of exposing his past consumption practices to law enforcement and other authority figures was used to terrorize him. This author has feared arrest, losing funding, being disqualified for professional licensure, being expelled from collegiate and professional training schools, and has feared for his loved ones being caught in harm's way for his actions. Given the extensive use of informants in drug law enforcement, not knowing whom or how much to trust someone has also been a source of mental distress for him. This author has also personally met individuals who were hunted and captured by law enforcement officials at local, county, state, and federal levels for their contraband biota-related activities. He has met people living with serious illnesses (e.g., rheumatoid arthritis, failed-back surgery syndromes, cancers, chronic pain) who have literally been terrorized, whose bodies have been tortured when incarcerated or pulled from organ transplantation lists, or forcibly denied access to therapeutic and palliative cannabis consumption or other medical treatment. He has met many patients in clinical scenarios who might benefit from the medicinal use of contraband biota with potential therapeutic utility, such as cannabis, and felt the frustration of being unable to offer or try these therapeutic options due to the illegal status of these substances. He has met people who were facing or have faced life sentences for their cannabis cultivation practices--even when that cannabis was being used for medical purposes. He has met others who have faced grave legal consequences and attendant distress such as lengthy incarceration or its threat related to their possession and consumption of other biotic psychoactive substances, such as Psilocybe fungi. This author is also familiar with many other cases that he has read about or learned about from trusted sources. In sharp relief to this, he has also met people who have complete amnesty and sanctuary from prosecution related to their contraband biota consumption or production practices. He has met the grower who produces cannabis for the United States federal government and who holds the patent on single-cannabinoid medicine marketed as a legal alternative to contraband cannabinoid botanicals. He has met three out of four of the ill and disabled American patients who, as a result of a landmark lawsuit, are supplied cannabis to consume by the federal government because their physicians attested to its profound therapeutic value for them, and he has also met chronically ill patients in Canada who have been granted amnesty by the Canadian government to produce and consume cannabis. Finally, he has met individuals in various cafes in Vancouver, British Columbia and Amsterdam, Holland who enjoy relative freedom in public spaces that grant them a sanctuary for cannabis and Psilocybe fungi consumption. The existence of such widely divergent scenarios of amnesty and terror helps to underscore the critical role the environment plays in producing or preventing mental distress related to contraband psychoactive biota consumption.

Applying a political ecology of mental distress approach can help to understand how individuals and groups react to such environments. The clash between localized understandings of particular human-environment interactions and medical and public policy interpretations of those same interactions creates stressful conditions to which individuals and groups adapt. The modern concept of human adaptation has its roots in the cultural ecological work of the mid-1950s spearheaded by Julian Steward, student of the renowned anthropologist Alfred Kroeber [13,14]. The geographer Bennett [15] in his book on the inhabitants of the Great Plains of North America, helped to bring the human adaptation concept into geography. He offers valuable insight to the nature and type of adaptation patterns that individuals and groups practice when responding to problems and stressors. He sees adaptive behaviors as coping mechanisms that take a multitude of forms including "problem-solving, decision-making, consuming and not consuming, inventing, innovating, migrating, staying" (p. 11). To define or measure adaptation, Bennett suggests looking in terms of goal-satisfaction and resource conservation (p. 13). He insists on making the very useful distinction between *adaptive strategies *and *adaptive processes*. Adaptive strategies pertain to "the pattern formed by the many separate adjustments that people devise in order to obtain and use resources and solve immediate problems" and are generally conscious decisions. Adaptive processes pertain to "changes introduced over relatively long periods of time by the repeated use of such strategies or the making of many adjustments" and usually can be seen only by outside observers (p.14). The study of human adaptation patterns is a significant part of work in human geography and has been examined in diverse contexts, from natural hazards and threats to subaltern studies of peasant resistance strategies.

Given the universal, embodied human experience of distress and threat, it should be no surprise that adaptation to various types of environmentally-induced distress may take similar forms. Mitchell [16], in a review on the geographic study of natural hazards, states as much: "the insights of natural hazard research may aid in developing general theories of man-environment relations. The possibility exists that models of human response to environmental threat may also function as analogs for research on man's adjustment to more pervasive forms of social stress" (p. 312). Medical geographer Mayer [17] also recognizes the relevance of socially stressful stimuli for a political ecology of disease framework: "it is important in the context of political ecology to ascertain the causes, both intentional and unintentional, of social isolation and marginalization" (p.451). It is additionally equally important to ascertain the responses and adaptations of individuals and groups to conditions that produce these sorts of social stress.

Literature in ecological anthropology, such as work by Vayda and McCay [18], has made significant headway in showing how the category of hazards can subsume "social and psychological insults" such as mental distress which produce demonstrable "psychological and behavior adaptations strategies." In their review of work in this area, they write broadly about the nature of various hazards that face organisms and groups and their responses. They are particularly concerned with those hazards that lead to "the risk of losing an 'existential game' in which success consists simply in staying in the game" (p.293). This aptly describes the hazards faced by those who produce and consume or otherwise come into close contact with contraband biota, such as cannabis and other forbidden biotic substances. Indeed, Vayda and McCay see the notion of 'hazards' to encompass not only "extreme geophysical events such as floods, frosts, droughts, hurricanes, and tornadoes" but also "predation by warfare, plundering or raiding...exactions of tribute and taxes...or acts of religious persecution" (p.294). Those affected by the psychoactive substance prohibitions under a policy commonly known as the 'war on drugs', variously referred to by its detractors as 'the pharmacratic inquisition' or 'psychopharmacological Calvinism' [19-22], do certainly perceive their life hazards with terms such as predations, raiding, and persecution. These hazards form the backbone of the adaptation pressures to consider in a political ecology of mental distress at facing possession-related legal problems.

## Substance-related disorders diagnostics and possession-related legal problems

The 'substance' nomenclature was first widely popularized as a result of sweeping, comprehensive, and international template-setting United States federal legislation passed by the Congress in 1970 and still in effect today. This legislation, known as the Controlled Substances Act, created a chapter under Title 21 "FOOD AND DRUGS" of the federal code: "CHAPTER 13 - DRUG ABUSE PREVENTION AND CONTROL." Note the clear and explicit language that identifies this as a public health-styled disease "prevention and control" regulatory schema. Under this policy, a system of five 'Controlled Substance' Schedules was created. In moving from Schedule V to Schedule I, increasing degrees of criminal prohibition apply, with Schedule I 'substances' falling for all practical purposes into the category of total prohibition (with exemptions granted for extremely limited medico-scientific research, religious use, and 'instruction'). Substances in Schedules V, IV, III, and II are allowed for progressively restricted medical use and research but are otherwise prohibited. According to the regulations, Schedules I and II apply when "The drug or other substance has a high potential for abuse." Biotic psychoactive substances appear only in Schedules I and II. They either appear directly by name (e.g., "Marihuana", "Peyote", "Opium poppy"), or by implied identification with a unique secondary metabolite made by the organism (e.g., "Psilocybin" referring to a metabolite made by 186 species of Psilocybe fungi). On an official government website, the name of the organism that produces the scheduled metabolite is listed alongside the chemical name [23]. To give an idea of size, currently 132 substances are listed in Schedule I, 62 in Schedule II, 31 in Schedule III; 70 in Schedule IV; and 10 in Schedule V--305 'controlled' substance in all [24]. These can be referred to as the 'Controlled 305'. In this vast controlled substance-scape, the focus of this paper is in on biotic psychoactive substances, which have a far more extensive history of human use and are far more easily studied with a political ecology lens compared to abiotic ones (though the two are surely interconnected). The number of distinct biological organisms represented in the 'Controlled 305' probably number in the low hundreds (with psilocybin-, dimethyltryptamine-, and related tryptamine-containing organisms making up the vast majority [25,19]. It is worth noting that several of the 305 substances appear endogenously in the human body, such as dimethyltryptamine [26] and morphine [27]. If one is charged for this internal possession of controlled substances or worried about it, perhaps one can mount the defense of 'guilt by association'! All absurdities aside, the final downstream target of this entire enforcement schema has to do with particular situations in which human bodies make close contact with one of these politicized plants, fungi, or chemicals, and the ensuing embodied experiences that follow as a result of the body's absorption of active chemicals into its bloodstream. Given this context, do these consumptive experiences amount to 'drug' or 'Substance' Abuse?

In the current fourth edition of the American Psychiatric Association's DSM (Diagnostic and Statistical Manual for Mental Disorders) [5], Substance-Related Disorders are divided into two groups: the Substance Use Disorders (Substance Dependence and Substance Abuse) and the Substance-Induced Disorders (Substance Intoxication, Substance Withdrawal, Substance-Induced Delirium, Substance-Induced Persisting Dementia, Substance-Induced Persisting Amnestic Disorder, Substance-Induced Psychotic Disorder, Substance-Induced Mood Disorder, Substance-Induced Anxiety Disorder, Substance-Induced Sexual Dysfunction, and Substance-Induced Sleep Disorder). Of these, the mental disorders that will be focused on here are the Substance Use Disorders, especially Substance Abuse but also to some extent Substance Dependence. Substance Intoxication disorders, which also merit attention, will not be addressed here due to space constraints.

To begin a brief modern history of the nosology of Substance Abuse, one must start in 1952, with the publication of the original DSM. There, Substance Abuse or drug abuse was listed as a Sociopathic Personality Disturbance--the same category that homosexuality was placed in (which was finally removed in 1973 but its "treatment" not fully repudiated until 1998 [28]. Both the DSM-I and DSM-II were virtually identical to the ICD (International Classification of Disease) nosology developed by the WHO (World Health Organization). The DSM-III, released in 1980, was a significant break from this; it incorporated approaches that were developed by researchers at Washington University School of Medicine during the 1970's. It introduced the multiaxial system of diagnostic evaluation. In this schema, Substance Abuse, as a class of Substance Use mental disorders, was classified under Axis I, which was reserved for syndromes such as depression and schizophrenia. For the first time, DSM-III classified Substance Use mental disorders in a separate diagnostic category distinct from the personality disorders. DSM-III-R (revised) was released in 1987, and in 1988, the most extensive process yet of reworking the Substance Use mental disorders section began. This reworking was completed 6 years later with the release of the DSM-IV in 1994. With regards to Substance Use disorders, the most significant change in the DSM-IV was the specific definition and clear enumeration of four free-standing, pathognomonic diagnostic criteria for Substance Abuse mental disorder, as distinguished from Substance Dependence mental disorder [29].

Stepping back for a moment, it appears that in the history of Substance Abuse nosology, there was a time in history when the psychopathological category of 'Substance Abuse' *itself *was on the chopping block, just barely escaping deletion during the period between the DSM-III and DSM-III-R. Schuckit [29] and Helzer [30], respectively, writing in the *DSM-IV Sourcebook*, relay the following bits of psychiatric lore:

The change between DSM-III and DSM-III-R represented an entire reorientation in the concept of abuse and dependence...the term *dependence *was broadened considerably. As a consequence, the framers of DSM-III-R originally proposed to delete the concept of abuse, feeling that the entire spectrum of substance-related problems was now incorporated into the broad concept of dependence. At the last minute, however, pressure from the field required that the term *abuse *be reinserted into the manual. However, abuse was now viewed as a residual diagnosis that was to be applied only to individuals who still had some substance-related difficulties but who did not fit into even a broad approach to dependence [29](p.7)

...

In a personal communication to the Substance Use Disorders Committee, Richard Frances recalled that there was an attempt to drop the term *abuse *in the DSM-III-R criteria, but that it was reinstituted at the time of the field trials by the popular demand of those attempting to use the new DSM-III-R criteria. [30](p.25)

Who might have been the most vocal opponents of the Substance Use Disorders Committee's planned deletion --the 'squeakiest' wheels? It is unclear. Nevertheless, this category of mental disorder known as 'substance abuse' has persisted, notwithstanding how ever so tenuously it survived near-deletion or protestations about the essentially pejorative nature of the diagnosis recorded in the *American Journal of Psychiatry *[31,32]. The question remains: how to go about characterizing it? A definition of substance abuse emerged by consensus when the question was posed to a panel of 99 substance abuse experts by Rinaldi and colleagues [33]. Using this Delphic approach, the expert panel concluded that 'drug abuse' is "any use of drugs that causes physical, psychological, economic, legal, or social harm to the individual user or to others affected by the drug user's behavior" (quoted in [30](p.24)). The current DSM-IV-TR (2000, TR = "Text Revision") definition of substance abuse, with its four free-standing criteria of distress or impairment manifestations accompanying substance use patterns--shirking of work/school obligations, engaging in physically hazardous behavior, recurring substance-related legal problems, and social/familial disputes--is essentially based on the panel's consensus definition. This four-criterion algorithm allows for 15 possible criteria combinations (1 only, 2 only, 3 only, 4 only, 1+2 only, etc.) that will satisfy the diagnosis for Substance Abuse. The focus of this paper's inquiry is only on the third diagnostic criterion for substance abuse mental disorder which describes persons engaged in a patterns of substance use who present "clinically significant...distress" "as manifested by...recurrent substance-related legal problems" which have "occurred repeatedly" or "been persistent" in the past year (Criterion A3). The DSM-IV states that if persistent or recurrent substance-related legal problems arise in conjunction with substance use, then that substance use pattern is maladaptive and a Substance Abuse mental disorder is the likely underlying diagnosable psychopathology that explains the person's "clinically significant...distress."

Rather than uncritically accepting this criterion as a factual description of psychopathy, the analysis here is directed towards potential depathologization of this criterion. Such an orientation follows the lead of numerous medical geographers in the field, such as Parr [34-36], Stock [37], Gesler [38], and Jones and Moon [39], who advocate the necessity of maintaining critical perspectives on highly socially-contingent disease-like states and giving due attention to alternative explanations for such states by patient-subjects. This paper attempts to question the basis of the A3 diagnostic criterion and depathologize the mental distress described therein on the grounds that additional, unaccounted social variables influence the manifestation of mental distress by some substance-related legal problems. Issues with this 'legal problems' criterion have, in fact, been raised by others in substance abuse and general medical literatures. For example, Alexander [40], in a paper in *The American Journal of Drug and Alcohol Abuse *that presents a "Marijuana Screening Inventory", notes some difficulties with criterion A3, in the case of Cannabis Abuse:

Subjective clinical judgment enters into Cannabis Abuse criterion distinctions regarding the meaning of 'recurrent' or 'maladaptive pattern.' For example, legal consequence risks are present with any marijuana use level, but may remain latent, or risk exposure only if a person drives or buys. Behavioral frequency cutoffs are not sufficiently clear regarding 'legal' or 'driving' problems with marijuana to allow consistent clinical agreement that a 'recurrent' 'maladaptive' pattern exists. (p.622)

Another commentator, Earleywine, a well-known academic psychologist who studies cannabis-related issues, writes in a response letter questioning the conclusions of a study published in the *Journal of the American Medical Association *about rising rates of cannabis abuse disorders in a particular urban population that "recurrent marijuana-related legal problems qualify users for the abuse diagnosis. Marijuana arrests increased dramatically in the decade studied (1991-2001)...which could account for the observed increases in the disorders" [41]. Earleywine's point rests on the necessity of establishing an analytically useful distinction between cannabis use disorders and cannabis arrests, showing that more aggressive enforcement of cannabis prohibition laws may better account for the "observed increases in the disorders", rather than any uptick in underlying incidence of psychopathology.

What is most problematic about the criterion is that the psychopathology-manifesting substance-related legal problems that the DSM-IV describes *include *those that arise from nonviolent, 'victimless infractions' of substance prohibition laws--in other words, legal charges or other legal problems related to the possession, production, and pharmacological delivery of contraband substances or discovered metabolic evidence of their consumption. For shorthand, these can be called substance-possession legal problems (with metabolites being a form of 'internal' possession). That such legal problems are also included in the criterion's assessment is absolutely indisputable as the manual specifically enumerates them. Shown in Table 1 is a comprehensive compilation of all the occurrences of the concept of "legal problems" in the DSM-IV, all of which appear in Substance-related disorders section of the manual with the sole exception of a single reference made to "legal difficulties" in the manual's description of conduct disorder. Italics have been added to highlight specific references to legal problems that arise from nonviolent infractions. Simply reading the italicized words brings into relief how these distressing legal problems, for the framers of the DSM-IV, translate into mental disorder.

**Table 1:** Substance Abuse Mental Disorders and Possession-Related Legal Problems in DSM-IV-TR.

*a*	***Substance Abuse **mental disorder*, *diagnostic Criterion *A3: "recurrent *substance-related legal problems *(e.g., arrests for substance-related disorderly conduct)"
**b**	**Alcohol Abuse (305.00) **mental disorder: "Legal difficulties may arise because of alcohol use (e.g., arrests for intoxicated behavior or for driving under the influence)."

***c***	***Cannabis Abuse (305.20) **mental disorder*: "...legal problems that *may occur as a consequence of arrests for cannabis possession*."

***d***	***Cocaine Abuse (305.60) **mental disorder*: "Legal difficulties *may result from possession or use of the drug*."

***e***	***Hallucinogen Abuse (305.30) **mental disorder*: "...legal difficulties *may arise due to behaviors that result from *intoxication or *possession of hallucinogens*."

***f***	***Amphetamine Abuse (305.70) **mental disorder*: "Legal difficulties *typically arise *as a result of behavior while intoxicated with amphetamines (especially aggressive behavior), *as a consequence of obtaining the drug on the illegal market, or as a result of drug possession or use*. Occasionally, *individuals with Amphetamine Abuse will engage in illegal acts *(*e.g., manufacturing amphetamines*, theft) *to obtain the drug*; however, this behavior is more common among those with Dependence."

**g**	**Inhalant Abuse (305.90) **mental disorder: "Users can also become agitated and even violent during intoxication, with subsequent legal and interpersonal problems."

***h***	***Opioid Abuse (305.50) **mental disorder*: "Legal difficulties *may arise *as a result of behavior while intoxicated with opioids or *because an individual has resorted to illegal sources of supply*."

***i***	***Phencyclidine Abuse (305.90) **mental disorder*: "Legal difficulties *may arise due to possession of phencyclidine *or to behaviors resulting from Intoxication (e.g., fighting)."

j	"The category of Substance Abuse does not apply to caffeine and nicotine";

k	"The term *abuse should be applied only to a pattern of substance use that meets the criteria for this disorder*; the term should not be used as a synonym for "use," "misuse," or "hazardous use";

l	"The essential feature of Substance Abuse is a maladaptive pattern of substance use manifested by recurrent and significant adverse consequences related to the repeated use of substances. *In order for an Abuse criterion to be met, the substance-related problem must have occurred repeatedly during the same 12-month period or been persistent*";

m	There may be recurrent substance-related legal problems (e.g., arrests for disorderly conduct, assault and battery, driving under the influence) (Criterion A3)";

n	"*Substance-Related Disorders are distinguished from **nonpathological substance use **(e.g., "social" drinking) and from the **use of medications for appropriate medical purposes **by *the presence of a pattern of multiple symptoms occurring over an extended period of time (e.g., tolerance, withdrawal, compulsive use) or *the presence of substance-related problems *(*e.g.*, medical complications, disruption in social and family relationships, vocational or financial difficulties, *legal problems*);

o	"Although a diagnosis of Substance Abuse is more likely in individuals who have only recently started taking the substance, *some individuals continue to have substance-related adverse social consequences over a long period of time without developing evidence of Substance Dependence*."

Italics added.

To recap, the codified, canonical diagnostic criteria found in the DSM-IV-TR that health care providers use to evaluate patients' substance consuming patterns for Substance Abuse disorder require providers to take careful note, ideally (but often not) in the course of a structured interview, of "clinically significant...distress". The DSM-IV-TR states that this "distress" and the "maladaptive" substance use pattern that led to it can be "manifested by...recurrent substance-related legal problems" which have "occurred repeatedly" or "been persistent" in the past year to qualify for the disorder. The idea is that because someone is engaging in a continuing behavioral pattern of substance use despite the adverse consequence of legal problems, s/he must be mentally disordered. The DSM-IV-TR diagnostic criteria for substance use disorders do not interrogate the substance control criminal sanction systems in which patients live; substance-related legal problems are never themselves seen as *the problem*. Under this rubric, one's experience of distress that is manifested by pending or year-long persisting legal problems is understood as mentally *disordered *in light of the ordinary and ubiquitous nature of the globalized contraband biotic substance prohibition enforcement regimes--i.e., the prevailing *order*. These regimes are understood to be naturalized and normalized aspects of the environment; for someone to run counter to them is understood as maladaptive, and any resultant distress is interpreted as a diagnostic sign of mental illness.

## Banning biota and sowing the seeds of distress

Medical anthropologists have long reminded medical social scientists to beware of slippage between pathology and expressions of cultural and social difference. Merrill Singer warned of this when he wrote: "the adaptationist perspective appears to assign inequities in social relationships to the environment, thereby not only legitimizing those inequities as natural, but implying that the noxious consequences of exploitation are indicators of the maladaptation of politically and economically subordinate groups" [14](p.226).

This paper's contention is that current medical thinking on substance abuse has acquiesced to what could be called 'drug war diagnostics'. Consider an alternate explanation to account for a substance-using patient's mental distress as manifested by recurrent or persistent biotic substance possession legal problems. What if their mental distress is a normal response to a system of substance/social control that has itself set up a maladaptive relationship with the psychoactive substance-replete global environment? If this may be the case, might it be unreasonable then to expect people to adapt to a system of biotic substance control committed to eradicating whole botanical species, not only from their personal lifeworlds, but also entirely from the face of the planet, save for a handful of authorized sites and personages?

**Figure 1:**
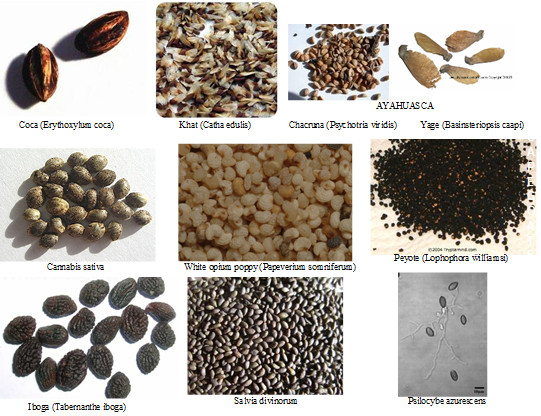
**Key Contraband Germplasms**. (from top left, rightward) Coca: http://web.archive.org/web/20061018053434/http://www.ethnogarden.com/cart/index.pl/catid_77/proid_292/_/_/CocaSeeds/ErythroxylumCoca, Khat: http://www.shamanica.com/Catha%20edulis.asp, Chacruna: http://web.archive.org/web/20061018155352/http://www.ethnogarden.com/cart/index.pl/catid_77/proid_250/_/_/Chacruna/PsychotriaViridis, Yage: http://www.shamanic-extracts.com/xcart/shamanic-products/banisteriopsis-caapi-seeds.html, Cannabis: http://www.cannabisculture.com/articles/4477.html, Opium: http://www.plantcultures.org.uk/plants/opium_poppy_traditional_medicine.html, Peyote: http://tryptamind.com/grow_peyote.html, Iboga: http://www.shamanic-extracts.com/xcart/shamanic-products/tabernanthe-iboga-seeds.html, Salvia Divinorum: http://www.sagewisdom.org/sdseeds.html Psilocybe: http://www.erowid.org/plants/mushrooms/mushrooms_cultivation_az2.shtml.

The following section of the paper will critically assess how this biotic substance control system spreads itself biogeographically and sociospatially at multiple scales, from a broad, global environmental level to the ultra-local perspective of the individual consumer. In the so-called "public health" campaign to prevent and control substances abuse, State governing bodies the world over have essentially extraprocedurally taken ownership of entire species of naturally occurring, pharmacologically active biota from the plant and fungal kingdoms--out of the hundreds of types of naturally occurring psychoactive biota--and criminalized their consumption outside of narrow, official channels. Ten species that evolved on Earth's biosphere are currently at the heart of this policy, through direct or indirect reference in international, federal or state-level Schedules. They are: *Papaver somniferum *L., *Erythroxylum coca *Lam, *Cannabis sativa *L., *Lophophora williamsii *J.M.C., 186 Psilocybe fungi spp., *Catha edulis *Vahl, *Tabernanthe iboga *L., *Banisteriopsis caapi *C.V.M. &*Psychotria viridis *Ruiz & Pav, and *Salvia divinorum *Epling & Játiva. More commonly, these are known as opium, coca, cannabis, peyote, mushrooms, khat, iboga, ayahuasca, and salvia. Of these, the first three--opium, cannabis, and coca--have the longest standing ownership-bans in the modern era with the most far-reaching consequences. These are in fact ownership-bans because global biotic psychoactive substance prohibitions grant legitimate, monopoly ownership of the biota--or, at root, select germplasms (plant genetic resources) (Figure 1)--wherever they may occur and at whatever generational age of the species--to State authorities while prohibiting safe access by others, literally bioimpoverishing unauthorized billions through force or the threat of force. Those who civilly disobey these regulations by consuming or facilitating consumption of contraband biota--possession law violators--are, in effect, stealing from world governments, hence, getting "busted", and many are routinely charged for such crimes. The institution of such bans on nature requires a historical act of biocolonization: a prior political call of species-wide, claim staking, i.e., a depletion of the commons pool of plant genetic resources through decree. It is this historical act that allows the past participle form of the verb 'control' in the phrase 'controlled substances' to assert itself as absolutely commonplace and normalized.

The points in space of interaction between *Homo sapiens *and these elements of banned non-human nature are points of material and sociocultural significance; their geographies are shaped by ecological and sociopolitical forces and thus easily lend themselves to the analytic frame of political ecology. When a human being comes into close contact with a banned botanical life form in her or his environment, experienced psychosocially at this most local scale is the rule of global scale international and national prohibition laws that encircle the botanical biota with boundaries which historically have been shaped by sociopolitical forces of power, influence, and authority--basic issues that concern political economy--that have the effect of alienating individuals from freely associating with these elements of the natural world. These are exactly the sorts of boundaries that Robbins [7] is referring to when he writes:

In recent history, powerful modern institutions and individuals ([e.g.,] environmental ministries, multinational corporations, corrupt foresters) have gained undue and disproportionate power by explicitly attempting to divide and police the boundaries between human and non-human nature, even while allying themselves and building new connections to the non-human world, leading to unintended consequences and pernicious results. In the process, resistance emerges from traditional, alternative, and progressive human/non-human alliances marginalized by such efforts (usually along lines of gender, class, and race) (p.213).

Contact with banned psychoactive biota is also ecologically mediated through the organic distribution of living species, mutual adaptation (e.g., health-related behavior), and co-evolution (e.g., selective cultivation), which influence how often and in what context human and non-human species will come into gross and "deep" consumptive contact, the latter understood through the logics of pharmacology, physiology and metabolism. It is readily apparent, then, that the overall effects of the consumption of banned biotic substances wherever they may occur locally, such as those related to psychoactivation, are never determined solely by material or biophysical forces alone; rather, agency, culture, context, and psychological set play equally vital roles.

Biogeographic State ownership and control of whole species of life in the service of substance abuse prevention and control has a qualitative policy parallel only in the arenas of biological weapons control and endangered species preservation. In the former category, unauthorized persons found in ownership or possession of entire species of life (or quasi-life) such as plague (*Yersinia pestis*), tularemia (*Francisella tularensis*), *Ebola *virus, or processed derivatives of these and other species are subject to criminal sanctions. In the latter arena, unauthorized persons found in ownership or possession of threatened or endangered species of life such as the Salt Creek tiger beetle (*Cicindela nevadica lincolniana*), the African violet (*Saintpaulia ionantha*), and the White Rhinoceros (*Ceratotherium simum*) are also subject to criminal sanctions. Exceptions are commonly granted in both cases, and criminal penalties are rarely delivered. Governments' exertion of authoritative biogeographic control as per their international treaty or convention obligations over potentially mass violence-causing biological agents and species threatened with extinction has not led massive civil/political unrest or strife, mainly because these policies do not undermine basic social goals of peace, development, and sustainability. In essence, there is no valued benefit to exposing people to highly virulent pathogens or to wiping out endangered species that is being undermined, although these prohibitions are balanced against the fulfillment of people's desires to own biological weapons for self-defense or people's desires to consume and possess endangered species for aphrodisia or sport.

On the other hand, the banning of ten biota out of the hundreds with psychoactive potential, while heavily and yet often duplicitously enforced, do not further the goals of public health and safety as they are purported to do. On the contrary, they have led, over the course of several decades, to a significant amount of corruption, chaos and instability (secondary to money laundering), structural violence, direct violence (secondary to black markets), morbidity (such as untreated problematic substance use and the significant spread of HIV and HCV due to needle sharing and inaccessible clean injection equipment), mortality (overdose deaths from unregulated products), lengthy mass incarceration (1 in 99 adults were incarcerated in the US at the beginning of 2008, with non-violent offenders being the majority and drug offenders held the longest), execution (including summary and extra-judicial), ecological harms (e.g., from aerial spraying of herbicides), educational harms (e.g., teaching misinformation, exaggerating rare and lurid sequelae of psychoactive substance use to frighten youth into abstinence, withholding lifesaving harm reduction knowledge), and opportunity cost globally [19,42-57]. At root, this is because bans on psychoactive botanical biota, regardless of whatever 'hidden agendas' may additionally be at work, undermine longstanding medicinal, cultural, and religious practices and unsuccessfully attempt to politically suppress what may well be an acquired universal human drive for psychoactivation through categorically forbidding natural substances and policing populations for compliance [58-60]. This policy, often called a 'war on drugs' or 'drug abuse prevention and control' is seen by those who bear its brunt as a low-grade, persistent, prisoner taking war on steeped in the ideology of pharmacologicalism in which some substances are allowed and encouraged for psychoactivation (e.g, tobacco, alcohol, caffeine, sugar, cacao) and others, such as those listed above, are forbidden. Through this ideology, which ultimately makes no distinction between psychoactive substances that are of biotic or abiotic origins, numerous substances such as the Controlled-305 in the United States Code have come under the globalized system of differential prohibition. Since human drives must prevail for life to go on, there will always be a demand for these officially prohibited substances as long as there is information available about their effects. By creating a regulatory vacuum, substance prohibitions essentially ensure that the drive to psychoactivate, which may well be established in future research, will be met by and large in the most exploitative and damaging manner--maximizing harm and minimizing benefit at both the population and individual levels. An earnest attempt at public health would at the very least reduce the harms associated with the consumption of psychoactive substances by ensuring that such substances are safely self-administered, made available through safe and regulated channels with known and unadulterated compositions, and that the public is given factual, evidence-based education about their effects.

It is only diplomats and politicians from a past era who have created this unique biotic constellation carved out with scientific botanical taxonomy--this biogeographic catalogue of ten different types of banned germplasm. That these germplasms are members of a common class is strictly historical artifact and not due to any natural grouping. Authority-holders' enactment of biotic prohibitions has created an il/legal natural geographic lifeworld mapping for nearly every world citizen in which whole species and subsubspecies of botanicals have become bounded up and encircled by prohibitionist-pharmacologicalist borders that were drawn without civic engagement or due process afforded to the most heavily affected populations. Each species so bounded has a unique ecology, a unique consumption-efficacy profile, and a unique environmental and human utilization history. Each encircling biotic prohibition inscribed around a natural species is a unique 'map feature' of an individual's lifeworld that presents distinct 'lost opportunities' for their utilization of that biota to fulfill part of their medicine and health care delivery, nutritional, religious, chemurgic, and/or safe psychoactivation needs--all remaining virtually inaccessible to law-abiding citizens and society at large who are taught 'thou shalt not unlawfully trespass' the extraprocedurally drawn boundary lines. The vast majority of citizens will not want to *openly *disobey these rules by crossing the boundaries for fear of arrest and associated penalogic social, civil, and bodily death threats--pain delivery--that is ongoing and virtually omnipresent. As a resultant adaptive strategy, nearly all boundary-crossing is done clandestinely under the cover of a 'black' or underground half-trillion dollar market (alone worth perhaps 10%+ of total global market exchange) [9] or through private non-commercial land use and exchange. More often than not, end substance consumers are far removed from the cultivation and ecological embeddedness of the biota they consume.

## Asserting the human right to health

It is those who are using biotic substances and are discovered or detected, possibly through acts of accidental indiscretion, and charged with violations of substance possession laws that are the focus of this inquiry. They have transgressed laws that purport to prevent and control, at the population level, the very mental disorder that they stand to be diagnosed with. Should not an attempt be made to distinguish bona fide psychopathology from transgressions of laws supposedly meant to prevent and control that psychopathology? Legal problems or not, do people have a right to consume biotic substances? This question has been generally explored from a legal perspective by Boire using ideas related to freedom of thought, or "cognitive liberty", but consideration vis-à-vis the human right to health is a little-explored approach [61]. Drug control within the UN system is technically subordinate to other higher order principles, such as the promotion of human rights [62]. The immediate-past United Nations Special Rapporteur on the Human Right to Health has highlighted "the indispensable role of health professionals in the promotion and protection of the right to health" [63]. The Committee on Economic, Social, and Cultural Rights (CESCR), a body of independent experts that monitors implementation of the International Covenant on Economic, Social and Cultural Rights by its State parties, was established by the United Nations Charter-created Economic and Social Council (ECOSOC) of the UN General Assembly under ECOSOC Resolution 1985/17 of 28 May 1985 to carry out the monitoring functions assigned to the ECOSOC. The Committee has acknowledged that the human right to health "is closely related to and dependent upon the realization of other human rights, as contained in the International Bill of Rights, including the rights to food, housing, work, education, human dignity, life, non-discrimination, equality, the prohibition against torture, privacy, access to information, and the freedoms of association, assembly and movement" [64]. The human right to health, as enumerated in international law, implies certain freedoms and entitlements such as "*the right to control one's health and body*...and the right to a system of health protection which provides equality of opportunity for people to enjoy the highest attainable level of health" (emphasis added) [64]. The current UN Special Rapporteur on the Human Right to Health, Anand Grover, has proposed a new international drug control framework grounded in the human right to health that would call for "assessment of the scientific evidence of a drug's effects on the individual and the public" and "the public health and human rights effects of each controlled drug." It additionally would allow for "traditional, cultural use of drugs, whose public health impact has been shown to be very limited, such as coca leaves in Bolivia and various forms of cannabis in India" [65]. It may well be argued that the right to determine food and drug preferences ought to be seen as a natural consequence of human dignity, especially vis-à-vis the human right to health, and the legitimate role of public policy ought to be harm minimization (as described above) and benefit maximization as related to these preferences [66]. The concept and term "benefit maximization" as a corollary and complementary goal to harm reduction in contemporary drug policy was originally articulated by Tupper, in reference to the globalization of ayahuasca drinking [67]. This should apply equally well to drugs or substances which are preferred for intoxication or other practices that are associated with psychoactivation. UCLA psychopharmacologist Ronald Siegel has written in his book *Intoxication: The Universal Drive for Mind-Altering Substances *[58] that

the medical purpose of intoxication is easier to understand if we think of intoxicating drugs as *adaptogens*. Technically, an adaptogen is a substance that helps people adjust to changes in their physical or psychological environments...Intoxicating drugs medicate the needs of the...drive for a change in state or mood...the pursuit of intoxication serves a legitimate medical purpose. The solution to the drug problems of our species begins when we acknowledge the legitimate place of intoxication in our behavior. (p.308-9)

Satisfying the putative acquired human drive for psychoactivation is a health issue and must be examined with ethics, reason, and patience--not with the usual hilarity, levity, and flippancy that dominates much discussion of this topic in the mainstream media, some policymaking circles, and countless casual conversations the first author has witnessed as a result of discussants' reliance on tropes from popular culture, memories of past embodied experiences or inclinations toward future sought out experiences of pleasure, and/or unexamined privileged positions of distance from the excesses of structurally violent drug enforcement regimes. To summarize, prohibitionist drug laws are, at root, a violation of the right to control one's health and body--essential pillars of the human right to health. Thus, in the authors' estimation, it is difficult to understand how the self-administration of any drug or substance per se can be understood as a criminal act; rather, this paper argues that the *criminalization *of drug consumption itself must be seen as an illegal act by States insofar as it violates their obligation to respect, protect, and fulfill the human right to health.

## An Alternative Framework: Harm Reduction and Benefit Maximization

What is readily apparent from a critical political ecology of disease perspective is that before a substance abuse mental disorder diagnosis can be made, patient-centered, subjectivist perspective demands scrutiny of the political context for patients' "substance-related problems". This would entail ethically interrogating the basis of the "legal" aspects of patients' problems, as well as seeking to uncover "hidden agendas" that may be at work [17](p.449). This paper argues that the success or failure of a so-called public health regulation like a substance abuse prevention and control law as it applies to a particular patient-citizen, i.e., whether or not he or she has distressfully transgressed the regulation, ought not to be the grounds on which a mental disorder diagnosis is made. Rather, the diagnosis of substance abuse mental disorder should be made based on whether or not the individual patient does indeed engage in problematic substance consumption practices. Just because the Substance Abuse prevention and control law, a supposed public health measure, has been flouted--with distressing consequences for the patient--does not mean that this is a sure sign that mental disorder is present in the patient. After all, how a patient-citizen's consumption practices came to articulate spatiotemporally with the public health regime of substance abuse disorder prevention and control to generate 'their' "legal problems" is not simply a function of their mental health. Depending on the effectiveness and sincerity of the public health regulation, regulatory transgressions may not be a sign of mental disorder, but rather one of governmental disorder. This possibility must be sincerely entertained, and the upcoming edition of the DSM should recognize this.

**Figure 2:**
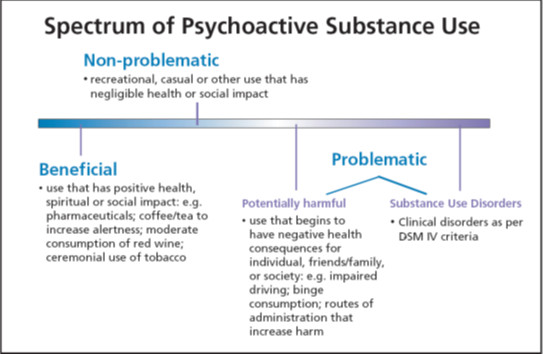
**Spectrum of Psychoactive Substance Use**. From "Every Door Is The Right Door: a British Columbia planning framework to address problematic substance use and addiction." May 2004.

An alternative approach to, for example, cannabis abuse diagnostics applied to a hypothetical patient-citizen in the United States based on the findings presented in this paper would be to jettison legal problems as a useful criterion to gauge cannabis abuse. Cannabis-related legal problems are unreliable indicators of psychopathology, not to mention often unjust [68]. It is better to focus on particular problems associated with an individual's cannabis consumption [69,70]. In fact, the whole substance use/abuse dichotomy ought to be discarded and the transition be made to a spectrum view, as has been adopted by the British Columbia Ministry of Health. In their framework for addressing problematic substance use [71], they include the diagram below (Figure 2) and note:

The Framework recognizes that instances or patterns of substance use occur along a spectrum from beneficial use to non-problematic use to problematic use (including potentially harmful use and substance use disorders). Substance use disorders represent the extreme and most damaging end of the spectrum. Some people choose to abstain from using psychoactive substances while some people choose to use only certain substances. It is important to emphasize that abstinence is a healthy lifestyle option. Nevertheless, many people choose to use substances and some do not develop serious problems because of this use. (p. 8)

Though they do not abandon the substance use disorders nosology in this particular model, the government of British Columbia takes an enlightened approach to understanding psychoactive substance use. Applying this to cannabis use, it is clear that cannabis consumption can be beneficial, non-problematic, or problematic for the consumer. Distinguishing between problematic and non-problematic use is straightforward: probe for the existence of medical/psychosocial problems, leaving legal issues aside as a Dutch health care provider would be inclined to do, given the Netherlands' system of *de facto *cannabis (re)legalization. If problems are identified, attention should be focused on reducing those particular harms associated with cannabis use for the patient-citizen. Distinguishing between non-problematic versus beneficial use of cannabis is more difficult, given the *relaxant *properties of cannabis use, and given consumers' tendency to reduce or substitute for alcohol consumption, which has its own health benefits. Perhaps this determination, if it must be made at all, ought to be done on strictly subjective grounds, as per "the new subjective medicine" that seeks to take "the patient's point of view" on matters related to health status and withdrawal of life-support [72]. Given that cannabis is not recognized as a medicine at the federal level and in 34 states, it is likely that consumers may not be 'looking' for medicinal or beneficial effects, though when doctors and patients do find them, they ought to be free to use them. A questionnaire that focuses on quality of life, stress reduction, spirituality, somaesthetics [73], self-directed psychotherapeutics, self-care, and related issues would likely help to elicit beneficial aspects of cannabis consumption that a consumer may only be dimly aware of on open-ended questioning.

Continuing with the cannabis example, one aspect of cannabis consumption that risks total neglect (and 'abuse', if you will) in substance use/abuse and related discourses is the relationship that human beings develop with environmental biota that they discover, produce and consume, such as plants, and in particular the cannabis plant. Appreciation, seed planting, nurturing, harvesting, and consumption of cannabis are all part of a human-environment relationship between two biotic species that both descended from a common evolutionary ancestor between 1 and 2 billion years ago [74,75]. Medical geographer Hester Parr, in her 2006 talk at the University of Washington Department of Geography Colloquium, spoke about the emotional benefits that mental patient-citizens glean through their experience with gardening and plant care. Her research showed that horticultural practices helped to "ground" patient-citizens. One respondent noted: "You slow your thoughts down to the speed of the plant and what's happening to it." Another said: "...you go into a sort of trance." A third said: "You can go into this place that is not you and it's not the world" (2006, first author's notes from lecture). While such reactions may not be specific to human relations with plants and may occur as a part of any slow or meditative activity, it is clear human-plant relationships can have cultural and therapeutic aspects to them. This side of cannabis consumption and production is totally neglected in modern 'use/abuse/dependence' discourses.

Problematic use of any and all of the "Controlled 305" substances--plus alcohol--can be referred to with the diagnosis of Substance Abuse mental disorder, effectively eliding their diverse pharmacology. A tremendous amount of confusion is created by this scattered grouping of 306 chemicals and organisms into a catch-all term of 'Substances', 'drugs', or the pejorative term, 'dope.' Frequently alcohol is distinguished from the rest with vapid phraseology such as "alcohol and drugs." With such terminology, it is easy to see how and why the most problematic aspects of use of certain 'substances' in the list of 305 Controlled Substances can become misattributed to use of any other particular 'Substance' in the classification. As this paper has attempted to show, the use of proper language is critically important in the arenas of substance regulation policy and substance-related diagnostics. The following is a quote from McGill University Law Professor Desmond Manderson's paper entitled the "Archaeology of Drug Laws" [76] that underscores the importance of using accurate language when discussing drug policy. Manderson examines the universal tone of ferocity and repulsion at ugliness that is betokened in drug laws in the twentieth century. He places the word 'narcotic', which appears in the 1914 Harrison Narcotic Act, the first punitive federal drug law in the United States, in its historical context when answering the question: "What is the effect of the endemic use of this word?"

It implies that the substances previously identified only as 'dangerous' are united in their medical and pharmacological nature as well as by their legal status. There is a patina of scientific legitimacy attached to that crucial word 'narcotics'. By using it, the title tells us to expect a certain kind of scientific substance to be dealt with. The frame gives medical legitimacy to the like treatment of the substances dealt with in the Act.

Clearly the language of the title is a nonsense: neither cocaine nor cannabis is a narcotic (i.e. sedative). By categorising them using a technical medical term, however, their legal treatment was shored up with scientific authority, all the while underscoring the belief that 'drug use' itself was a medical problem. 'Narcotics' in the first place gives the illusion of a scientific basis to legal policy and, second, presents the drug question as a medical rather than a moral issue. The word acts as a legitimation and a defense of government intervention. Here, then, we see the power of the language of the title to construct a reality, to expropriate authority by the use of persuasive words, and to redefine a social event--the consumption of cannabis, for example--by placing it within a frame so that it becomes seen to be scientifically dangerous and medically unjustifiable.

The language of narcosis, however, while it reflected and effected a focus on the medical dangers of drug use alien ...was, by the 1970s, no longer an adequate description and justification of people's fears...by [then]...the concern over drug use...[was]...to do partly...with the non-medical or recreational use of drugs...The drug user may not be suffering from any medical problem but he or she is nevertheless 'abusing' drugs. In fact, the power of the language comes exactly from the intentional conflation of use with misuse and abuse.

## Conclusion

Moving into a post-'drug war' era, society will need a fuller understanding of the penal pain inflicted en masse by the current system per banned substance. In order to maximize consumer-related health protection and safeguards in public policy while at the same time realizing their fullest potential in medicine, each of the ten banned botanical species will require a separate medical geographic treatment through the lens of the political ecology of health and disease, as each presents unique health justice policy issues and challenges. The human-environment relationships surrounding each will require 'daylighting', a concept borrowed from urban design and planning which normally refers to a process by which an underground stream is redirected into an above-ground channel where it is visible by the light of day. In the context of biotic substance use, daylighting means the application of scholarly labor so that the light of understanding is shone on underground human-environment relationships which are presently in the dark and out of view. For example, with coca, a longstanding Andean medicinal and sacramental plant, comes issues related to the concentration and isolation the alkaloid cocaine, which occurs naturally as 0.1% by weight of the leaf, and its conversion to crack cocaine with the addition of baking soda (sodium bicarbonate) and heat. Additionally, with opium, also an invaluable medicinal plant with cross-cultural roots, comes issues related to the concentration and isolation of morphine, which is about 10% by weight of dried poppy juice, and its conversion to heroin (diacetyl morphine) with the addition of dry vinegar (acetic anhydride) and heat. A political ecology of health must necessarily attend to these concentrates and the contexts in which they are produced from mature botanicals, distributed in an underground economy, and consumed. As far as biotic substance use-related mental distress manifested by possession-related legal problems is concerned, the critical political ecology of disease approach applied here has been successful in depathologizing this mental distress and seeing it instead as a product of a structurally violent substance abuse prevention policy gone too far, undermining fundamental human-environment biotic relations and the human right to health.

## Competing interests

The authors declare that they have no competing interests.

## Authors' contributions

SKA conceived of the study, conducted it, and drafted the manuscript. GTC, CZ, RM, MS, and JDM participated in the study's design and coordination and were involved in manuscript drafting revision. All authors read and approved the final manuscript.
